# Baseline Demographic and Clinical Characteristics of Patients with Adrenal Incidentaloma from a Single Center in China: A Survey

**DOI:** 10.1155/2017/3093290

**Published:** 2017-08-07

**Authors:** Lele Li, Guoqing Yang, Ling Zhao, Jingtao Dou, Weijun Gu, Zhaohui Lv, Juming Lu, Yiming Mu

**Affiliations:** ^1^Department of Endocrinology, Chinese PLA General Hospital, Beijing 100853, China; ^2^Chinese PLA Key Laboratory of Endocrinology and Metabolism, Beijing 100853, China

## Abstract

**Aim:**

To investigate the clinical and endocrinological characteristics of patients with adrenal incidentaloma (AI).

**Materials and Methods:**

This retrospective study enrolled 1941 AI patients hospitalized at the Department of Endocrinology, Chinese PLA General Hospital, Beijing, China, between January 1997 and December 2016. The patient gender, age at visits, imaging features, functional status, and histological results were analyzed.

**Results:**

Of the 1941 patients, 984 (50.70%) were men. The median age was 52 years (interquartile range: 44–69 years). 140 cases had bilateral AI. Endocrine evaluation showed that 1411 (72.69%) patients had nonfunctional tumor, 152 (7.83%) had subclinical Cushing syndrome (SCS), and 82 (4.33%) had primary hyperaldosteronism. A total of 925 patients underwent operation for removal of 496 cortical adenomas (53.62%), 15 adrenal cortical carcinomas (1.62%), and 172 pheochromocytomas (18.59%). The bilateral group had a higher proportion of SCS (18.57% versus 7.10%, *P* < 0.001, *P* = 0.006). A mass size of 46 mm was of great value in distinguishing malignant tumors from the benign tumors, with sensitivity of 88.2% and specificity of 95.5%.

**Conclusions:**

We reported the baseline demographic and clinical characteristics of patients with AI in a large series from a single center in China.

## 1. Introduction

An adrenal incidentaloma (AI) is a previously unsuspected adrenal mass discovered on an imaging study performed for an unrelated reason. Increased use of computed tomography (CT), ultrasonography, and magnetic resonance imaging (MRI) has led to its frequent discovery. Current estimates of the prevalence of AI ranges in various studies: 2.3% at autopsy, 4% in retrospective CT series, and 5.8% in oncological studies; its prevalence reportedly increases with aging [1–3]. As AI is increasingly recognized in current medical practice, such masses raise challenging questions for both physicians and their patients and represent one of the leading reasons for endocrinological consultation [4].

The term AI is not a single entity, rather it is an “umbrella” definition comprising a spectrum of different pathological entities that share the same path of discovery [5]. Only malignant or hyperfunctional AI (excess production of cortisol, aldosterone, and catacholamines) needs active treatment; thus, it is mandatory to identify such patients. A number of clinical series regarding AI have been reported in the literature [6–11]; however, high heterogeneity among data sources has been noted, and there is no international consensus regarding the management of AI. Moreover, studies of AI in the Chinese population are insufficient. Therefore, we conducted a retrospective analysis of the clinical and endocrinological characteristics of patients with AI at a tertiary referral hospital in China to provide greater insight into their work-up and management.

## 2. Materials and Methods

This retrospective study was conducted at the Department of Endocrinology, Chinese PLA General Hospital, Beijing, China. The Chinese PLA General Hospital Ethics Committee specifically approved this study, and written informed consent was obtained from all patients.

According to the definition of AI, we excluded patients with the following: (1) clinical symptoms relevant to the adrenal gland; (2) abnormal biochemical results, that is, severe paroxysmal hypertension, hypokalemia, and clinical signs of hypercortisolism or hyperandrogenism, indicating abnormal adrenal function prior to initial radiological investigation; or (3) previous history of malignancies. However, patients with adrenal metastasis as the initial discovery of a malignancy were included. Patient information was obtained retrospectively from their medical records by use of a specifically tailored questionnaire. All completed questionnaires were individually checked for inconsistencies before statistical analysis, and double inclusion of the same patient was avoided. Finally, 1941 eligible patients hospitalized between January 2006 and December 2016 were enrolled.

After history and physical examination, all patients underwent biochemical evaluation to assess their functional status. Biochemical evaluation consisted of measurements of patients' 24 h urine-free cortisol level (2 times); serum cortisol and plasma ACTH levels at 0:00, 8:00, and 16:00, followed by overnight dexamethasone test (DST, 1 mg dexamethasone given at 23:00); and measurement of serum cortisol and plasma ACTH levels at 8:00 the next morning (<50 nmol/L was considered normal). Patients with post-DST cortisol levels ≥50 nmol/L underwent additional tests, specifically the low-dose DST (LDDST, 4 mg/48 h; cutoff level, 50 nmol/L). Patients with post-LDDST cortisol levels ≥ 50 nmol/L were diagnosed as having subclinical Cushing syndrome (SCS). Patients with an aldosterone-rennin ratio (ARR > 20) underwent any of the following three confirmatory tests to definitively confirm or exclude PA: saline infusion test, captopril challenge test, and postural stimulation test. Pheochromocytoma was diagnosed on the basis of the concentrations of catecholamines and metanephrines in two 24 h urine samples and adrenal CT or MRI scan before operation and confirmed or corrected according to the histological analysis. The diagnosis of CAH was based on clinical history, current clinical status, and hormonal criteria (mainly 17-OHP) and confirmed by *CYP21A2* gene mutational analysis. After ruling out the above status, a diagnosis of nonfunctional adrenal tumor was established.

All the hormones in the study were analyzed by chemiluminescence immunoassay. Cortisol and ACTH levels were analyzed by chemiluminescence immunoassay. ACTH was detected using an Immulite 2000 Analyzer (Siemens Healthcare Diagnostics Inc., LA, USA). Cortisol was measured with an ADVIA Centaur Analyzer (Siemens Healthcare Diagnostics, Tarrytown, NY, USA). The CT imaging technique was not standardized because of the different hospitals that patients initially visited, and only CT-estimated mass location and size were considered for statistical analysis. Histological diagnosis was reported according to the original description, with a central review of pathological specimens.

Statistical analysis was performed using SPSS Software (Version 17.0). Values are expressed as median (interquartile range) or as numbers with percentage. Categorical data such as gender and clinical/radiologic features were compared using *χ*^2^ test. Group data with a normal distribution were compared using a *t*-test. A two-sided *P* value  < 0.05 was considered to indicate statistical significance.

## 3. Results

### 3.1. General Characteristics of Patients with AI

**Figure 1:**
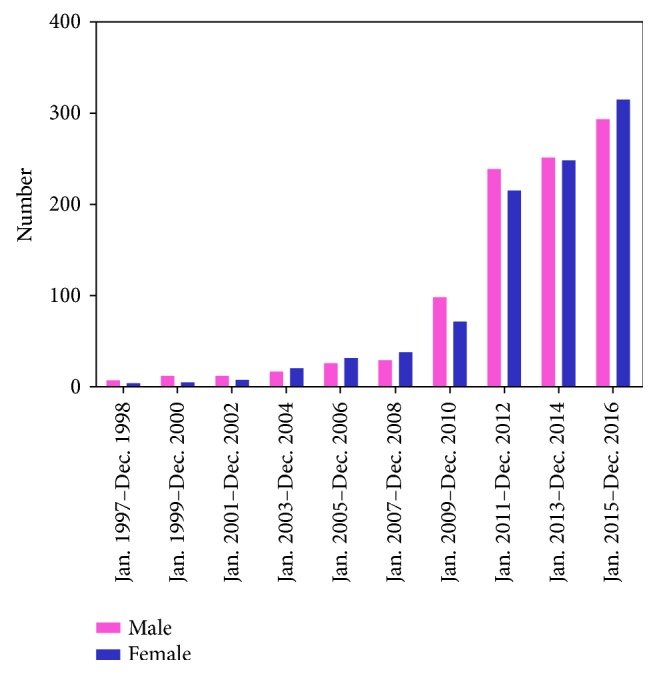
The distribution of patients according to the year.

**Figure 2:**
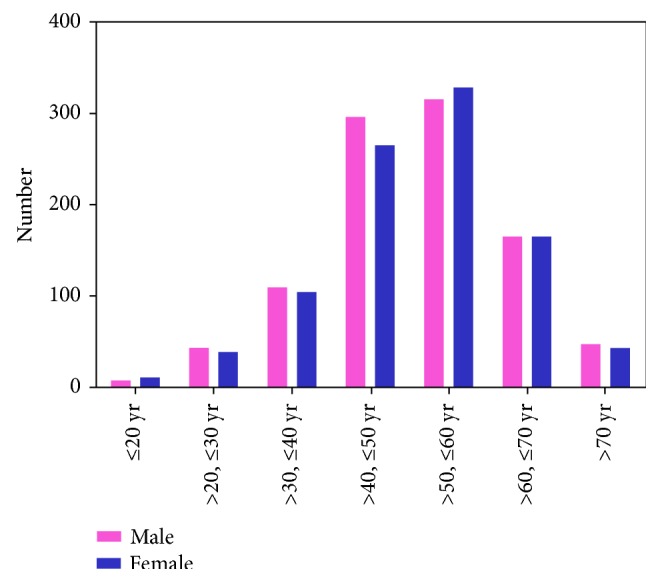
The distribution of patients according to age.

Between January 2006 and December 2016, 1941 patients eligible were enrolled. The distribution of patients according to the year is shown in Figure 1, which shows that the number of cases gradually increased each year. The demographic characteristics are presented in Table 1. Of the 1941 patients, 984 (50.70%) were men. The median age of patients was 52 years (interquartile range, 44–69 years), and the distribution of patients according to age is illustrated in Figure 2: the highest proportion of AI was in patients aged 50–60 years. Overall, unilateral tumors were detected on the left side in 865 (46.93%) cases and on the right side in 838 (45.47%); the remaining 140 cases had bilateral AI. Hypertension was observed in 1045 (53.9%) patients, diabetes in 257 (13.3%) patients, dyslipidemia in 576 (29.8%) patients, fatty liver in 786 (45.04%, 1742 patients with data available) patients, and obesity (body mass index [BMI] > 28 kg/m^2^) in 514 (26.48%) patients. Routine medical checkup was the main method (42.14%) for detection of clinical onset leading to disease discovery. The predominant complaint included low back pain (7.41%) and abdominal pain (13.80%).

### 3.2. Functional Status and Histological Results of Patients with AI

Endocrinological evaluation showed that 1411 (72.69%) patients had nonfunctional tumor, 152 (7.83%) had subclinical Cushing syndrome (SCS), 82 (4.33%) had primary hyperaldosteronism, and 227 (11.69%) had pheochromocytomas. Of the 561 patients with available 17-OH-progesterone (17-OHP) data, 4 were confirmed to have congenital adrenal hyperplasia (CAH). A total of 925 patients underwent operation for removal of 496 cortical adenomas (53.62%), 15 adrenal cortical carcinomas (1.62%), 172 pheochromocytomas (18.59%), 45 paragangliomas/ganglioneuromas (4.86%), 54 cysts (5.85%), and 41 myelolipomas (4.43%).  Table 2 presents the detailed functional status and histological results of the patients.

### 3.3. Clinical Characteristics and Functional Status of Patients with Bilateral and Unilateral AI

As shown in Table 3, patients with bilateral and unilateral AI did not differ in terms of median age and BMI; however, the gender difference was statistically significant between the two groups (*P* = 0.001). The bilateral AI group had a higher proportion of male patients than the unilateral AI group. The patients' functional status showed that the bilateral group had a higher proportion of patients with SCS (18.57% versus 7.10%, *P* < 0.001) and a lower proportion of patients with pheochromocytoma (4.29% versus 11.92%, *P* = 0.006) than the unilateral group. The proportion of nonfunctional tumor and PA showed no differences between the two groups.

### 3.4. Clinical Characteristics and Tumor Types by Size

**Figure 3:**
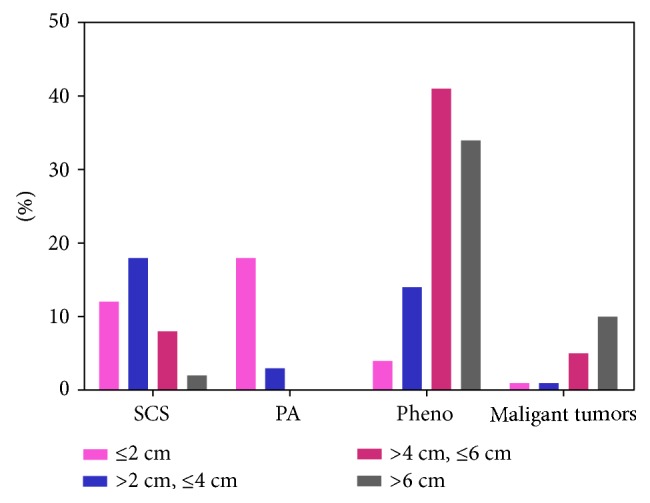
Proportion of each type of tumor according to the size. Malignant tumors include adrenal cortical carcinoma, metastatic carcinoma, lymphoma, and sarcoma. SCS: Subclinical Cushing syndrome; PA: primary hyperaldosteronism; Pheo: pheochromocytoma.

**Figure 4:**
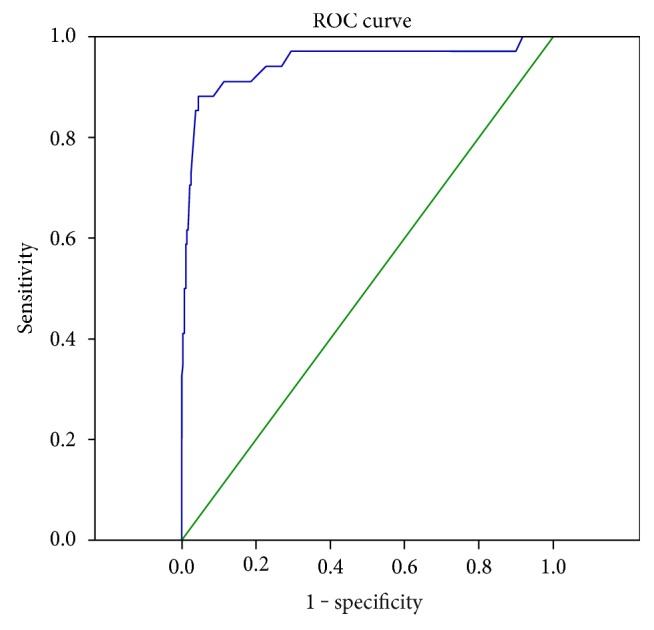
Area under the receiver-operating characteristic curves for the diagnosis of malignant and benign tumors.

Of the 925 operated patients, 828 had data available on the mass size. On the basis of the widest diameter of the largest tumor, these cases were classified into the following groups: ≤2 cm (*N* = 159), 2–4 cm (*N* = 828), 4–6 cm (*N* = 398), and >6 cm (*N* = 127). The clinical characteristics and tumor types are presented in Table 4. The highest proportion of SCS, PA, and pheochromocytomas was observed in the 2–4 cm, ≤2 cm, and 4–6 cm groups, respectively. In the >6 cm group, the proportion of malignancy increased sharply. The proportion of each tumor type according to mass size is presented in Figure 3. Receiver-operating characteristic (ROC) analysis was performed to evaluate the diagnostic value of mass size for distinguishing between malignant and benign tumors. The area under the curve (AUC) was 0.946 (95% confidence interval: 0.892–1), and the optimal cutoff value was 46 mm, with a sensitivity of 88.2% and specificity of 95.5%, shown in Figure 4.

## 4. Discussion

To the best of our knowledge, this is the largest clinical series of AI reported from a single center in the literature. AIs are being detected with an increasing frequency due to the widespread increase in cross-sectional imaging and is gradually emerging as a common clinical problem. Our data show that 1562 (80.47%) patients hospitalized between January 2011 and December 2016 had AI, indicating an increasing rate of detection and greater awareness of AI.

Age distribution of our series of patients was wide, and the median age at visits was 52 years, which is in line with the findings of previous studies [8, 10, 12]. The increasing age of the general population and a trend towards more advanced investigations in the elderly maybe have contributed to the high detection rate in this age group. Alternatively, the high detection rate could be explained by an increased occurrence of cortical nodules with age, as observed in unselected autopsy series [13, 14]. This finding may represent a compensatory growth in response to the local ischemic damage of arteriosclerotic disease [15]. Mantero et al. [10] and Tabuchi et al. [8] reported a female tendency in their study, but our case series did not report significant gender differences; this could be partly explained by a referral bias, as nonfunctional adrenal adenomas occurred with comparable frequency in men and women in autopsy series [13, 14]. In the present study, we found that the frequency of AI in both sides was comparable, and this finding is consistent with the recent literature published in 2013 and 2016 [6, 8, 16]; previous studies have shown contrasting results [10, 17]. These discrepancies might be due to the advances in medical imaging technology in recent years: ultrasonography was the diagnostic technique used in the previous series, and the right adrenal gland was better visualized by ultrasound than the left gland [18]. Therefore, recent advances in CT technology might have improved the detection of small tumors, especially in the left adrenal gland.

Clinically, upon discovery of AIs, two issues arise: functionality and malignancy. Regarding the functionality of masses, we observed that 1411 (72.69%) patients had nonfunctional tumors. Among the functional tumors, pheochromocytomas (11.69%) were the most frequently observed in our series, followed by SCS (7.83%) and PA (4.22%). In the literature, the frequency of pheochromocytomas ranged between 1.5 and 23%, whereas that of SCS varied from 1.2% to 12% [5]; such a great variability could be attributed to the inclusion criteria and referral pattern of various studies. Furthermore, analysis of the hormonal data collected in our series confirmed that an endocrine work-up in patients with AI led to the detection of a remarkable number of subclinical hormone-producing tumors, which further addresses the importance of endocrine evaluation.

**Figure 5:**
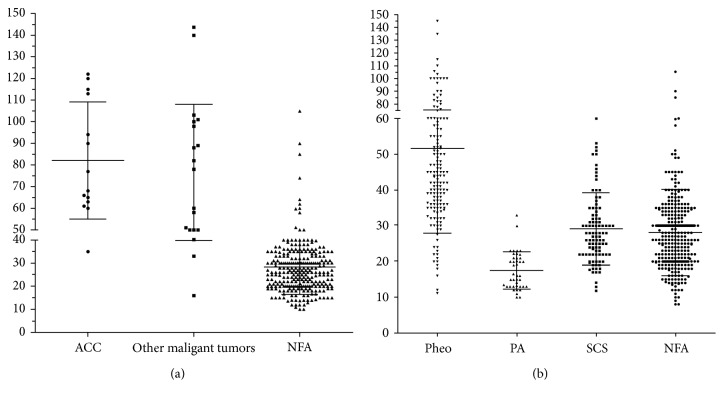
(a) The individual sizes of different functional tumors. (b) The individual sizes of benign and malignant tumors. Other malignant tumors include metastatic carcinoma, lymphoma, and sarcoma. SCS: subclinical Cushing syndrome; PA: primary hyperaldosteronism; Pheo: pheochromocytoma; ACC: adrenal cortical; carcinoma.

Bilateral lesions were detected in 7.6% of cases in the present series. Compared to patients with unilateral lesions, a higher frequency of SCS and a lower frequency of pheochromocytomas were detected in patients with bilateral lesions. Other series have also reported similar results [19, 20]. Moreover, previous studies indicated the probability that SCS is positively correlated with mass size [19]; this finding persisted when patients with unilateral AI were analyzed separately from those with bilateral AI [21, 22]. Our series showed that the highest proportion of SCS, PA, and pheochromocytomas were observed in 2~4 cm, ≤2 cm, and 4~6 cm groups, respectively.  Figure 5(a) shows the individual size values of different functional tumors. With regard to the precise assessment of SCS caused by bilateral lesions, previous studies assumed that the adrenal mass size in patients with unilateral AI was comparable with that of the largest mass in those with bilateral lesions and that in most patients with bilateral adrenal masses and SCS, one side of the adrenal gland, the larger side, is hypersecreting. All the above evidences support the importance of the mass size in the evaluation of its functional status. Notably, 3 of the 4 patients diagnosed with CAH initially presented with massive bilateral incidentalomas. In recent years, it has become evident that both homozygous and heterozygous patients with CAH have a high prevalence of AI [23–25]. In addition, although rare, some patients with AI were confirmed to have nonclassical CAH. The diagnosis of CAH in patients with AI is mandatory, as the patients are at risk for adrenal crises. The latest AI guidelines issued by the European Society of Endocrinology and European Network for the Study of Adrenal Tumors have recommended screening of CAH in patients with bilateral AI [26]. If AI is suspected clinically, assays for cortisol, adrenocorticotropic hormone (ACTH), dehydroepiandrosterone sulfate (DHEAS), and 17-OHP should be performed.

Regarding the malignancy of the masses, 37 of the 828 operated patients were confirmed to have malignant tumors by a final histological analysis. In the >6 cm group, the frequency of malignancy increased sharply. The size of individual benign and malignant tumors is shown in Figure 5(b). Overall, cortical adenomas were the smallest lesions, whereas malignant tumors were the largest lesions, despite some overlap. These findings confirm that the risk of malignancy is related to mass size. Previous studies suggest that nearly all lesions measuring <4 cm are benign and <2% are malignant [7, 27]. In the present study, ROC analysis was performed to evaluate the diagnostic value of mass size to distinguish between malignant and benign tumors; the results showed an AUC of 0.946 and optimal cutoff value of 46 mm, with a sensitivity of 88.2% and specificity of 95.5%. In China, overtreatment of nonfunctional AI measuring <4 cm is frequent due to a lack of agreement on this entity. Here, we suggest that tumors in this group, which are defined as having a low risk of malignancy by the imaging criteria, are generally not surgically resected. In addition to imaging characteristics, hormonal measurements could aid in the differential diagnosis because a great elevation of adrenal androgen levels, and in particular, DHEAS, could indicate the presence of primary adrenal malignancy. Thus, comprehensive analysis of imaging features and endocrinological results might be of remarkable clinical significance.

There are several limitations to our study that should be discussed. First, this was a retrospective review of cases from a single center. Second, some data were unavailable, which decreased the power of analysis. Finally, there was a lack of clinical and biochemical follow-up of the patients.

## 5. Conclusion

We reported the baseline demographic and clinical characteristics, especially the functionality and malignancy, of patients with AI in a large series from a single center in China. A total of 72.69% patients were diagnosed with nonfunctional tumors. Among the functional tumors, pheochromocytomas (11.69%) were the most frequently observed, followed by SCS (7.83%) and PA (4.22%). Bilateral AI was detected in 7.6% of the study population, and a higher proportion of SCS and a lower proportion of pheochromocytomas were seen in these cases as compared to the unilateral AI cases. Mass size was of great value in distinguishing malignant and benign tumors.

## Figures and Tables

**Table 1:** Baseline demographic and general characteristics of patients with adrenal incidentaloma.

Characteristic	Value
Gender, male/female, *N*	984/957
Age, median (IQR), y	52 (44, 59)
BMI, median (IQR), kg/m^2^	25.63 (23.19, 28.06)
≤24 kg/m^2^, *N* (%)	578 (29.78)
>24, ≤28 kg/m^2^, *N* (%)	849 (43.74)
>28 kg/m^2^, *N* (%)	514 (26.48)
Location, *N* (%)^a^	
Left	865 (46.93)
Right	838 (45.47)
Both	140 (7.60)
Mass size, median (IQR), mm^b^	23 (16, 36)
Surgery, *N* (%)	925 (47.65)
Concomitant disease, *N* (%)	
Diabetes mellitus	257 (13.3)
Hypertension	1045 (53.9)
Dyslipidemia	576 (29.8)
Fatty liver^c^	786 (45.04)
Reasons for initial imaging, *N* (%)	
Routine medical checkup	818 (42.14)
Low back pain	144 (7.41)
Abdominal pain	268 (13.80)
Urinary tract disease	81 (41.73)
Respiratory system disease	133 (6.85)
Hepatobiliary disease	80 (4.12)
Others	417 (21.43)

^a^1843 patients with data available. ^b^Widest diameter of the largest tumor among 1736 patients with data available. ^c^1742 patients with data available. IQR: interquartile range; BMI: body mass index.

**Table 2:** Functional status and histological results of patients with adrenal incidentaloma.

Functional status	*N* (%)	Histological result	*N* (%)
Nonfunctional tumor	1411 (72.69)	Adrenal adenoma	496 (53.62)
Subclinical Cushing syndrome	152 (7.83)	Pheochromocytoma	172 (18.59)
Primary hyperaldosteronism	82 (4.22)	Paragangliomas/ganglioneuromas	45 (4.86)
Pheochromocytoma	227 (11.69)	Adrenal cortical carcinoma	15 (1.62)
Adrenal cortical carcinoma	25 (1.29)	Cyst	54 (5.84)
Congenital adrenal hyperplasia	4^a^	Myelolipoma	41 (4.43)
Unknown functional status	40 (2.06)	Schwannoma	10 (1.08)
		Hyperplasia	15 (1.62)
		Metastatic carcinoma	8 (0.86)
		Others	69 (7.46)^b^

^a^561 patients with 17-OHP data available. ^b^Including hematoma, teratoma, sarcoma, hemangioma, lymphangioma, and lymphoma, 20 patients operated without histological results.

**Table 3:** Clinical characteristics and functional status of patients with bilateral and unilateral adrenal incidentaloma.

	Unilateral (*N* = 1703)	Bilateral (*N* = 140)	*P* value
Gender, male/female, *N*	852/851	86/54	0.010
Age, median (IQR), y	52 (44, 59)	55 (49, 62)	0.484
BMI, median (IQR), kg/m^2^	25.56 (23.14, 28.04)	26.64 (24.39, 28.86)	0.283
Mass size, median (IQR), mm	22 (15, 36)	20 (12, 29)	0.041
Surgery, *N* (%)	828 (48.62)	46 (32.86)	<0.001
Functional status, *N* (%)			
Nonfunctional tumor	1254 (73.63)	97 (69.29)	0.263
Subclinical Cushing syndrome	121 (7.10)	26 (18.57)	<0.001
Primary hyperaldosteronism	73 (4.29)	5 (3.57)	0.686
Pheochromocytoma	203 (11.92)	6 (4.29)	0.006
Adrenal cortical carcinoma	24 (1.41)	0	—
Congenital adrenal hyperplasia	1	3	—
Unknown functional status	27	3	—

IQR: interquartile range.

**Table 4:** Clinical characteristics and tumor types categorized by size in 828 operated patients.

	≤2 cm (*N* = 159)	>2, ≤4 cm (*N* = 398)	>4, ≤6 cm (*N* = 144)	>6 cm (*N* = 127)
Gender, male/female, *N*	79/80	181/217	80/64	66/61
Age, median (IQR), y	51 (45, 59)	51 (44, 58)	47 (37, 56)	44 (34, 53)
BMI, median (IQR), kg/m^2^	25.65 (23.38, 27.94)	25.85 (23.12, 28.40)	24.26 (22.06, 26.58)	24.42 (21.19, 26.64)
Type of tumor, *N* (%)				
Nonfunctional adenoma	84 (52.83)	189 (47.49)	21 (14.58)	10 (7.87)
Subclinical Cushing syndrome	19 (11.95)	70 (17.59)	11 (7.64)	2 (1.57)
Primary hyperaldosteronism	29 (18.24)	12 (3.02)	0 (0)	0 (0)
Pheochromocytoma	7 (4.40)	57 (14.32)	59 (40.97)	43 (33.85)
Paragangliomas/ganglioneuromas	2 (1.26)	14 (3.52)	11 (7.64)	13 (10.24)
Myelolipoma	2 (1.26)	12 (7.55)	12 (8.33)	12 (9.45)
Cyst	5 (3.14)	25 (6.28)	11 (7.64)	4 (3.15)
Adrenal cortical carcinoma	0 (0)	1 (0.25)	1 (0.69)	12 (9.45)
Other malignant tumors	1 (0.63)	2 (0.50)	7 (4.86)	13 (10.24)
Others	10 (6.3)	15 (3.77)	11 (7.64)	19 (14.96)
